# Knowledge, attitude, and adherence to malaria control guidelines and the prevalence of *Plasmodium* species infection in localities across transmission and ecological zones in Cameroon

**DOI:** 10.3389/fpubh.2023.1060479

**Published:** 2023-04-27

**Authors:** Livinus N. Tangi, Marcelus U. Ajonina, Marcel N. Moyeh, Hanesh F. Chi, Vincent N. Ntui, Pilate N. Kwi, Eric C. T. Toussi, Mary Progress S. Fung, FohTella Fah, Joel M. Mayaba, Franklin T. Formilack, Veronica N. Ntasin, Theobald M. Nji, Emmanuel V. Yenshu, Eric A. Achidi, Alfred Amambua-Ngwa, Tobias O. Apinjoh

**Affiliations:** ^1^Department of Microbiology and Parasitology, University of Buea, Buea, Cameroon; ^2^Department of Biochemistry and Molecular Biology, University of Buea, Buea, Cameroon; ^3^Department of Chemical and Biological Engineering, The University of Bamenda, Bambili, Cameroon; ^4^Department of Sociology and Anthropology, University of Buea, Buea, Cameroon; ^5^Department of Accounting, The University of Bamenda, Bambili, Cameroon; ^6^Medical Research Council Unit, The Gambia at London School of Hygiene and Tropical Medicine, Fajara, Gambia

**Keywords:** knowledge, attitude, practice, adherence, *Plasmodium* infection

## Abstract

**Background:**

Despite a scale up of control interventions over the years, malaria remains a major public health and economic concern in Cameroon, contributing considerably to hospitalization and deaths. The effectiveness of control strategies depends on the extent of adherence by the population to national guidelines. This study assessed the influence of human knowledge, attitudes, and practices related to malaria and its control on the prevalence of malaria parasite infection, with implications for the elimination of the disease.

**Methodology:**

This is a cross-sectional community and hospital-based study, covering the five ecological and three malaria transmission zones in Cameroon. A pre-tested semi-structured questionnaire was used to document socio-demographic and clinical parameters as well as knowledge, attitudes, and practices toward malaria control and management. Consenting participants were screened for malaria parasite with rapid diagnostic test (mRDT) of the peripheral blood. Association between qualitative variables was determined using the chi-square test and logistic regression analysis.

**Results:**

A total of 3,360 participants were enrolled, 45.0% (1,513) of whom were mRDT positive, with 14.0% (451/3,216) and 29.6% (951/3,216) having asymptomatic parasitaemia and malaria, respectively. Although most participants knew the cause, symptoms, and control strategies, with 53.6% (1,000/1,867) expertly knowledgeable about malaria overall, only 0.1% (2/1,763) individuals were fully adherent to malaria control measures.

**Conclusion:**

The risk of malaria in Cameroon remains high, with the population considerably knowledgeable about the disease but poorly adherent to national malaria control guidelines. Concerted and more effective strategies aimed at improving knowledge about malaria and adherences to control interventions are necessary to ultimately eliminate the disease.

## Introduction

Malaria remains a significant global public health and socio-economic burden, contributing to over 234 million infections in Africa, equivalent to 95% of global cases (1, 2) and 96% of deaths, accounting for the death of a child every 2 min in sub-Saharan Africa (sSA) (1, 2). This high incidence of the disease in Africa and particularly in the sSA region may be due to several factors, including poverty, inadequate knowledge of the disease interventions, and poor medical infrastructure (3–5). Furthermore, malaria is considered as a disease of rural areas, but factors linked to rapid, uncontrolled and haphazard urbanization, semi-urban and rural constructions, population flux as well as discrepant and inconsistent implementation of malaria control measures are increasing transmission in cities across Africa (6, 7).

Initiatives to eliminate the disease have witnessed diverse outcomes in regions and communities across the globe, with some 40 countries declared free of indigenous malaria after a 70-year effort in scaling down the disease prevalence from 30 million cases in the 1940s (8). The situation in sSA remains preoccupying, with the region constituting a reservoir for seeding resurgence and spread of the disease. Moreover, the decline in yearly incidence has stalled in the past couple of years with evidence of parasite and vector evading disease control measures (9).

Cameroon is one of eleven countries in the African continent that accounts for 70% of all malaria cases globally (9, 10). The country can be stratified into three zones of varying malaria transmission intensity, with the majority (71%) of its population residing in high and perennial transmission settings. Variation in intensity of transmission, human population flux, ineffectiveness and divergence in interventions against malaria are crucial determinants in sustaining the *Plasmodium* parasite and transmission of the disease. In fact, malaria has been on a rise in Cameroon since 2016, with about 6 million cases and 11 thousand deaths occurring annually, and children under the age of 5 years accounting for around 60% of cases and deaths (11). Morbidity rose from 25.9% in 2016 to 28.0% in 2018, while mortality increased from 14.3 to 18.3% during the same period (11).

Measures to wade off the disease in the country were initiated since 2002 and implemented by the National Malaria Control Programme (12). These strategies include free distribution of long lasting insecticide-treated nets (LLINs), indoor residual spraying, larviciding, diagnosis by light microscopy and rapid diagnostic tests, treatment with artemisinin-based combination therapy (ACT), free intermittent preventive treatment for pregnant women and infants, free treatment of uncomplicated malaria in children under 5 years old and seasonal malaria chemoprevention for children aged 3–59 months in the Far North and North regions (11). Due to these measures, prevalence decreased from 41% of the population reporting at least one malaria case episode in 2000 to 24% in 2018 (11, 13). However, the disease has been on the rise since 2016 despite these efforts (11).

Nevertheless, there is reported high variability in disease endemicity between geo-ecological settings, with prevalence of *Plasmodium* parasitaemia varying from 7 to 85% in children aged 6 months to 15 years after the use of long-lasting insecticide treated bednets (14, 15) and an increased prevalence reported between 2014 and 2017 (16). Furthermore, a relative decline in the therapeutic efficacy of some artemisinin-based combinations from 97% in 2006 to 90% in 2016 has been reported in the country (15). Moreover, the parasite population may also be adapting to human host immune pressure and mosquito vector environment. This could engender novel strains which are undetected in certain interventions such as diagnosis using the rapid diagnostic test (17–19).

These, in addition to population and individual behavior and practices contribute to compounding the fight against malaria in the country. Regular quantitative assessments of knowledge, practices, and behavioral patterns of populations in endemic communities is therefore essential to identify risk factors for malaria management (12) and refine control and elimination strategies.

Strategies for combating malaria have shown limitations over time, partly due to inadequacies in policies, commitment of populations to interventions, political and financial resources. Human behavior is thought to be an important determinant of the outcome of malaria control interventions (11). Some of the behavioral variables associated with malaria control include irregular, partial or none use of LLINs, self-diagnosis, and treatment, non-respect of schedule for IPTp and IPTi, and poor environmental sanitation (20).

Understanding how and why individuals/populations manifest different tendencies toward the management of malaria is essential in probing into reasons of failure in achieving breakthrough in the fight against malaria and devising novel implementation strategies toward eliminating the disease.

This study sought to investigate the relationship between the local people's awareness of the cause, symptoms and ways of protecting against malaria, their attitudes and practices toward adhering to control measures stipulated by the National control program of Cameroon and the risk of infection with the parasite. The findings will provide additional basis for redefining malaria control strategies and interventions.

## Materials and methods

### Ethical considerations

Ethical clearance was obtained from the Cameroon National Ethics Committee while administrative authorizations were sought from the relevant regulatory authorities in all areas where participants were enrolled. Only individuals or their guardians who volunteered to participate by signing a written informed consent, after adequate sensitization were enrolled. Assent was also obtained from all children above 12 years of age and pregnant women below 16 years of age in line with national gynecological guidelines.

### Study design and sampling sites

**Figure 1:**
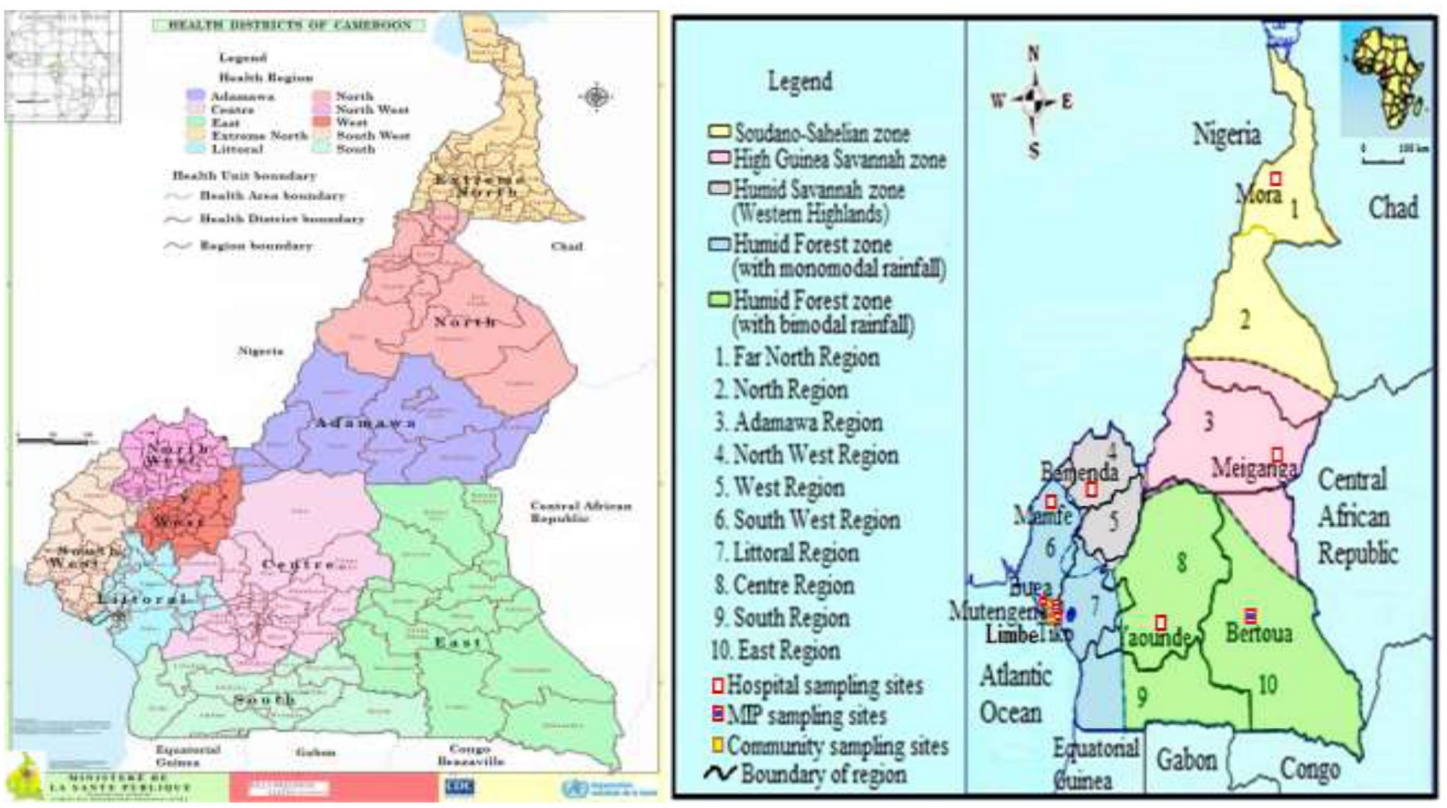
Map of ecological and malaria transmission zones in Cameroon (21, 22), indicating community/hospital sampling sites and Health Districts. https://dhis-minsante-cm.org/portal/index.html.

This is a descriptive cross-sectional community—and hospital—based survey conducted between April 2019 and October 2021 during the dry and rainy seasons across the five ecological zones of Cameroon. Sampling sites included communities and/or health facilities in and around the following towns—Buea, Limbe, Mamfe, Mutengene and Tiko of the Southwest Region and health facilities in Meiganga in the Adamawa, Yaounde in the Center, Bertoua in the East, Mora in the Far North and Bamenda in the Northwest Region with distinct characteristics (Figure 1; Table 1). Cameroon is located in Central Africa between latitude 7° 22′11″ North and longitude 12° 20′ 41″ East, with substantial geographic- and bio- diversity, across deserts, sea coasts, mountains, rainforest and savannah (23). *Plasmodium* species transmission in the country is markedly heterogeneous, with high and perennial parasite transmission occurring in the forest, coastal and humid savannah areas and seasonal transmission in the highlands and Sahelian dry savannah areas. Entomologic inoculation rates (EIR) are as high as 100 infective bites per person per month in the forest, coastal and humid savannah areas and 10 infective bites per person per month in the highlands and Sahelian dry savannah areas (24, 25). *Plasmodium falciparum* is the main malaria parasite species in the country while *Anopheles gambiae* is the predominant vector. Genetic structure studies of *P. falciparum* population suggested high diversity of 158 circulating strains in Cameroon (26–28). Seventy-one percent of the approximately 25 million inhabitants in the country live in high transmission regions (13).

**Table 1:** Features of the study sites across the five ecological zones.

**Characteristics**	**Ecological zones**				
	**Sudano-Sahelian**	**High Guinea Savannah**	**Humid Savannah**	**Humid Forest (Monomodal rainfall)**	**Humid Forest (Bimodal rainfall)**
Region(s)	Far North	Adamawa	Northwest	Southwest	Center, East
Town(s)	Mora	Meiganga	Bamenda	Buea, Limbe, Mamfe, Mutengene, Tiko	Bertoua, Yaounde
Temperature (°C)	15–40	12–34	15–28	17–34	17–33
Season	Two: short rainyand long dry	Two: long rainy and short dry			Four: two rainy and two dry
Humidity (%)	49–80	78–83	56–86	82–87	48–86
Precipitation (mm)	610–760	1,500–1,533	2,400–3,000	3,033	1,357–1,540
Rainfall (mm)	500−1,200	1,200–1,600	2,000–3,000	3,000–4,000	1,600–2,000
Malaria transmission	Seasonal	Low	High and perennial		
EIR (Infective bites/person/ month)	10		100		
Main ethnic group(s)	Sudanese		Semi-Bantu	Bantu & Semi—Bantu	Bantu
Major religion	Islam		Christianity		
Major activities	^*^Cattle rearing, Commerce, Farming, ^#^Fishing, Public service, Schooling				

^*^Cattle rearing is common in the Sudano-Sahelian, High Guinea Savannah & Humid Savannah.

^#^Fishing is a major activity in the towns of Limbe and Tiko in the Humid Forest ecological zone with monomodal rainfall. Temperature,^1^ humidity and precipitation,^2^ and rainfall (21).

Data from the demographic and health surveys and from the malaria indicator survey, revealed vegetation and altitude as important predictors of the geographical distribution of malaria in Cameroon (29). The main ethnic groups include Sudanese (Foulbe) in the Adamawa and Northern regions, Bantu in the South, Center, East, Littoral and part of the Southwest regions, and Semi-Bantu in the Northwest, West and part of the Southwest regions. The predominant occupations include commerce, farming, education and state/private service workers.

### Study population and sample survey

Hospital facilities were randomly selected in each ecological zone among the main public health facilities serving the town while communities were first identified as rural and semi-urban and then randomly selected. The population was then sensitized on the project objectives, methods and possible benefits/risks through health personnel, elites and/or community leaders. Participants were then invited to a central enrollment point and only resident adults interviewed in either English/French (national languages) or pidgin (local language) were recruited. A minimum of 317 participants per target cluster was required based on standard sample size formula (30), assuming a 29% prevalence of malaria in Cameroon as reported previously (31).

### Data acquisition and definition of terms

A pre-tested semi-structured questionnaire was used to document information about socio-demographics, knowledge about malaria and attitudes/practices in the management of the disease. Each participant's axillary temperature was measured using a digital thermometer, with fever defined as body temperature ≥ 37.5°C taken during the survey. Knowledge of malaria refers to the ability to name the agent or vector responsible for the disease or its transmission, any of the signs/symptoms, or any strategy applied to protect oneself from infection with or transmission of the agent.

Adherence to malaria control was defined as practice(s) that conform to current national and global guidelines for preventing infection and transmission, diagnosis, and treatment of malaria. An ITN was defined as any long-lasting insecticidal net, any bednet factory-treated with insecticide and obtained <36 months ago, or any bednet treated with insecticide <36 months ago (14). ITN use was defined as reportedly sleeping under a bednet or ITN the previous night. Ethnic identity, people who share common socio-cultural characteristics and are of the same ancestry (32), was defined as the self-reported ethnic group of the mother (or father if the mother's could not be obtained). Parasitaemia was defined as presence of parasite antigens in blood using a malaria rapid diagnostic test. Clinical malaria was defined as fever or history of fever together with parasite antigen and asymptomatic parasitaemia as parasite infection in the absence of fever or history of fever. Localities were classified as rural, semi-urban and urban based on the national administrative designation, population size as well as the presence or absence of infrastructure and services as described (33, 34).

### Blood collection and processing

Capillary blood samples were collected from each participant following a finger prick under standard aseptic procedure. *Plasmodium* infection status was ascertained using PfHRP2/pLDH malaria rapid diagnostic kit (SD Bioline^TM^, Alere, South Korea) and results interpreted following manufacturer's instructions. Briefly, about 5 μl of sample from each participant was placed in the sample window of the RDT cassette and three drops of diluent added. The results were then read after 15 min, with the presence of two (or three), one or no distinct line indicative of a positive, negative or invalid result, respectively. Hemoglobin (Hb) concentration was determined using a hemoglobinometer (Hangzhou Sejoy Electronics, Hangzhou, China) according to the manufacturer's instructions and anemia defined as Hb <11.00 g/dl of blood (14).

### Statistical analysis

Data were double entered in Microsoft Excel 2016 and analyzed using SPSS Statistics 20.0 (IBM Corp, Atlanta, GA, USA). Association between proportions were explored using the Pearson's Chi square (*x*^2^) or logistic regression. For multivariate analysis, the variables were selected based on statistical significance in the univariate models and only variables of known clinical relevance or with *p*-value <0.25 in univariate analysis were included in multivariate models. Results were reported as adjusted odd ratios (OR) together with their confidence intervals. To avoid duplication or repeated observation from parent/guardian's response on behalf of their children, only responses for individual's 15 years and above were analyzed for knowledge, attitude and practices against malaria. Statistical level of significance was set at *P* ≤ 0.05 at a 95% confidence interval.

## Results

### Characteristics of the study population

A total of 3,360 participants were enrolled in this study from Urban (44.4%), rural (43.4%) and Semi-Urban (12.3%) settings in all five ecological zones of Cameroon. Majority of the participants were enrolled from community surveys (62.5%), the Humid Forest zone with monomodal rainfall (84.3%), Semi—Bantu ethnic group (65.6%), aged 21−59 years (47.7%), females (60.9%, 2,045), resident at low altitude (64.8%) and had household sizes of 3–5 (45.7%) (Table 2). More than half of the population surveyed had fever or reported history of fever (55.3%) while 39.3, 29.6, and 14.0% had anemia, malaria and asymptomatic malaria parasitaemia, respectively at enrolment (Table 3). The socio-demographic and clinical characteristics were significantly (*p* < 0.001 each) different between participants enrolled across the different localities of residence. Only participants aged 15 years and above were considered for further analysis about knowledge, attitude and practices in the management of malaria and their relationship with malaria parasitaemia.

**Table 2:** Socio-demographic characteristics of the study population (*n* = 3,360).

**Parameter**	**All participants [% (*n*)]**	**Locality [% (** ***n*** **)]**			***P*-value**
		**Rural**	**Semi-urban**	**Urban**	
**Socio-demographic characteristics**					
**Survey type**					
Community	62.5 (2,100)	92.9 (1,353)	74.5 (307)	29.5 (440)	<0.001
ANC	12.9 (434)	1.6 (24)	10.0 (41)	24.7 (369)	
Clinical	24.6 (826)	5.5 (80)	15.5 (64)	45.7 (682)	
**Age group (years)** ^*^					
<5	12.7 (422)	13.9 (203)	18.7 (77)	9.7 (142)	<0.001
5–9	13.4 (447)	15.9 (231)	14.1 (58)	10.8 (158)	
10–14	9.9 (329)	12.2 (178)	10.0 (41)	7.5 (110)	
15–20	10.5 (349)	6.7 (98)	10.0 (41)	14.3 (210)	
21–59	47.7 (1,591)	44.6 (649)	39.6 (163)	53.1 (779)	
≥ 60	5.9 (197)	6.7 (97)	7.8 (32)	4.6 (68)	
**Ecological zones**					
Sudano-Sahelian	2.6 (86)	0.0 (0)	0.0 (0)	5.8 (86)	<0.001
High Guinea Savannah	5.1 (181)	0.0 (0)	0.0 (0)	12.1 (181)	
Humid Savannah	3.1 (103)	0.5 (7)	6.3 (26)	4.7 (70)	
Humid Forest (Bimodal rainfall)	4.7 (159)	0.0 (0)	0.0 (0)	10.7 (159)	
Humid Forest (Monomodal rainfall)	84.3 (2,831)	99.5 (1,450)	93.7 (386)	66.7 (995)	
**Altitude of residence**					
Low (≤200 m)	64.8 (2,177)	95.0 (1,384)	10.9 (45)	50.2 (748)	<0.001
Intermediate (>200 and ≤600 m)	13.8 (465)	2.7 (39)	76.0 (313)	7.6 (113)	
High (> 600 m)	21.4 (718)	2.3 (34)	13.1 (54)	42.3 (630)	
**Gender**					
Male	39.1 (1,315)	46.0 (670)	35.9 (148)	66.7 (994)	<0.001
Female	60.9 (2,045)	54.0 (787)	64.1 (264)	33.3 (497)	
**Ethnic identity** ^**^					
Bantu	25.5 (823)	27.9 (406)	15.5 (63)	25.8 (354)	<0.001
Semi-Bantu	65.6 (2,121)	66.9 (972)	84.2 (342)	58.8 (807)	
Sudanese	5.9 (191)	0.2 (3)	0.2 (1)	13.6 (187)	
Non-Cameroonians	3.0 (97)	5.0 (73)	0.0 (0)	1.7 (24)	
**Household size (persons)** ^***^					
≥10	5.1 (156)	4.0 (57)	6.9 (28)	5.7 (71)	<0.001
6–10	36.7 (1,127)	35.9 (511)	38.7 (156)	36.8 (460)	
3–5	45.7 (1,406)	46.0 (654)	43.9 (177)	46.0 (575)	
1–2	12.6 (386)	14.1 (200)	10.4 (42)	11.5 (144)	

ANC, Antenatal Clinic; Some variables had missing values ^*^3,335, ^**^3,232, ^***^3,075.

**Table 3:** Clinical characteristics of the study population.

**Parameter**	***N***	**All [% (*n*)]**	**MIP [% (** ***n*** **)]**						**Clinical [% (** ***n*** **)]**						**Community [% (** ***n*** **)]**					
			***N***	**All**	**Rural**	**Semi-urban**	**Urban**	***P*** **-value**	***N***	**All**	**Rural**	**Semi-urban**	**Urban**	***P*** **-value**	***N***	**All**	**Rural**	**Semi-urban**	**Urban**	***P*** **-value**
Anemia	2,920	39.3 (1,148)	331	62.8 (208)	70.8 (17)	64.1 (25)	61.9 (166)	0.678	615	41.8 (257)	54.1 (50)	46.8 (29)	37.5 (178)	<0.001	1,974	34.6 (683)	37.1 (461)	21.8 (67)	36.4 (155)	<0.001
Fever (during survey)	3,176	27.5 (872)	395	40.8 (169)	45.8 (11)	20.5 (8)	42.8 (142)	0.025	715	17.1 (523)	78.5 (62)	67.9 (38)	72.9 (423)	0.376	2,066	9.1 (188)	9.4 (126)	5.1 (15)	10.9 (47)	0.022
Fever history	3,180	47.2 (1,500)	415	24.6 (102)	37.6 (9)	19.5 (8)	24.3 (85)	0.253	666	89.6 (597)	92.4 (73)	82.5 (52)	90.1 (472)	0.124	2,099	38.2 (801)	40.8 (552)	29.6 (91)	36.0 (158)	0.001
Febrile (survey or history)	3,216	55.3 (1,780)	403	50.6 (204)	62.5 (15)	36.6 (15)	51.5 (174)	0.096	736	95.0 (699)	95.0 (76)	90.5 (57)	95.4 (566)	0.229	2,077	42.2 (877)	45.0 (603)	31.5 (95)	41.2 (179)	<0.001
mRDT positives	3,360	45.0 (1,513)	434	61.5 (267)	17.8 (17)	48.8 (20)	62.3 (230)	0.015	826	68.0 (562)	78.8 (63)	78.1 (50)	65.8 (449)	0.013	2,100	32.6 (684)	36.6 (495)	33.9 (104)	19.3 (85)	<0.001
Asymptomatic parasitaemia	3,216	14.0 (451)	403	25.8 (104)	29.2 (7)	31.7 (13)	24.9 (84)	0.592	736	2.2 (16)	2.5 (2)	4.8 (3)	1.9 (11)	0.315	2,077	15.9 (331)	17.3 (232)	21.9 (66)	7.6 (33)	<0.001
Malaria prevalence	3,216	29.6 (951)	403	34.2 (138)	41.7 (10)	17.1 (7)	35.8 (121)	0.042	736	63.0 (464)	76.3 (61)	7.3 (46)	60.2 (357)	0.005	2,077	16.8 (349)	19.5 (261)	12.3 (37)	11.8 (51)	<0.001
Antimalarial use prior enrolment	3,091	8.2 (255)	414	3.6 (15)	0.0 (0)	2.4 (1)	4.0 (14)	0.514	647	19.0 (123)	29.9 (23)	22.2 (14)	17.0 (86)	0.021	2,030	5.8 (117)	5.8 (76)	2.8 (18)	7.8 (33)	0.018

MIP, Malaria in pregnancy.

### Knowledge of malaria

**Figure 2:**
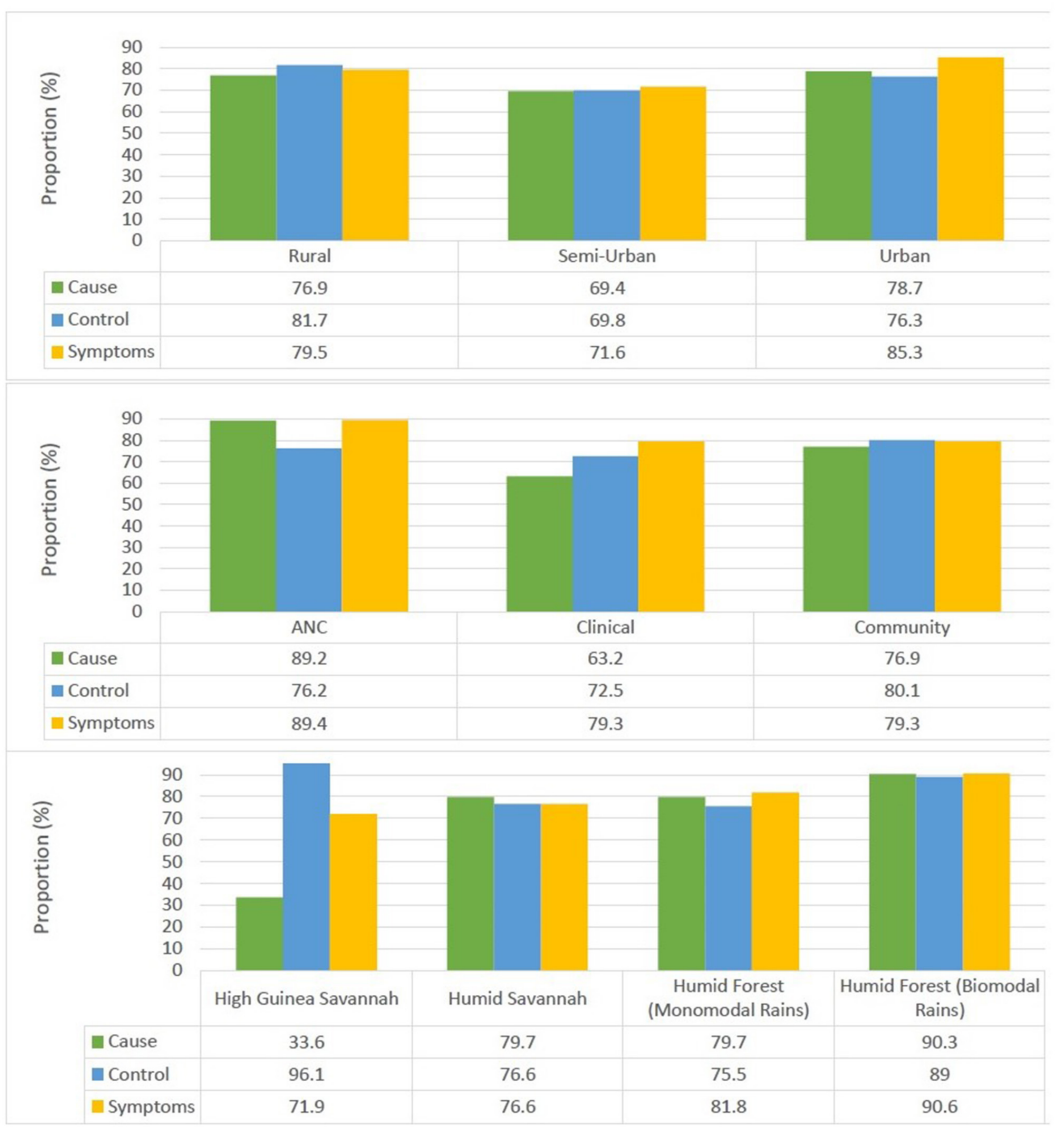
Knowledge of the cause, control measures, and symptoms of malaria in Cameroon.

More than three quarters of participants older than 15 years surveyed had knowledge of the cause 76.8%, symptoms 81.1%, and malaria control measures 77.8% (Figure 2). There was a significant association between the locality of residence and knowledge about the cause (*p* = 0.011), symptoms (*p* < 0.001) and control measures (*p* < 0.001). Individuals from urban settings were most knowledgeable about the cause and symptoms of the disease while their rural counterparts were most knowledgeable about control measures.

There was also a significant association between the survey type and knowledge of the cause (*p* < 0.001), symptoms (*p* < 0.001) and control measures (*p* = 0.004). Individuals enrolled from antenatal clinics were most knowledgeable about the cause (89.2%) and symptoms of malaria (89.4%) while those enrolled from community surveys were most knowledgeable about control measures (80.1%).

Furthermore, there was a significant association between knowledge of the cause of malaria and the ecological zone (*p* < 0.001), gender (*p* = 0.004), age group (*p* < 0.001), and ethnicity (*p* < 0.001) of the participant. Individuals from the Semi-Urban settings 69.4%, High Guinea Savannah zone 33.9%, males 73.1%, ≥60 years of age (51.7%) and Sudanese ethnic group (33.3%) were least aware of the cause of malaria.

In addition, knowledge of malaria control measures was also associated with the ecological zone (*p* < 0.001), locality of residence, age group (*p* = 0.001) and ethnicity (*p* < 0.001) of the participant. Inhabitants from Semi-Urban settings (69.8%), Humid Forest zone with monomodal rainfall (75.4%), aged 15–20 years (69.4%) and Bantu ethnic group (73.8%) were least knowledgeable about malaria control measures.

There was also a significant association between knowledge of malaria symptoms and the ecological zone (*p* = 0.033), gender (*p* < 0.001), age group (*p* < 0.001) and ethnicity (*p* = 0.002) of the participant. Individuals from the Semi-Urban settings (71.7%), High Guinea Savannah zone (72.5%), males (76.1%), ≥60 years of age (66.9%) and Sudanese ethnic group 73.4% were least knowledgeable about the symptoms of malaria.

The extent of knowledge about malaria was classified as none, limited, moderate or expert if the participant was reportedly knowledgeable about zero, one, two or all three factors, respectively. There was a significant association (*p* < 0.001) between the extent of knowledge about malaria and locality of residence, with 3.3, 11.7, 31.4, and 53.6% having no, limited, moderate and expert knowledge about the disease, respectively, overall (Table 4).

**Table 4:** Extent of knowledge of malaria in Cameroon (*n* = 1,867).

**Extent of knowledge**	**All participants**	**Locality [% (** ***n*** **)]**		
	**[% (** ***n*** **)]**	**Rural**	**Semi-urban**	**Urban**
None (0 factor)	3.3 (61)	3.5 (29)	7.9 (18)	1.7 (14)
Limited (1 factor)	11.7 (219)	10.9 (90)	18.3 (42)	10.7 (87)
Cause (C)	3.2 (60)	2.3 (19)	3.9 (9)	3.9 (32)
Control (K)	5.5 (103)	6.3 (52)	10.9 (25)	3.2 (26)
Symptoms (S)	3.0 (56)	2.3 (19)	3.5 (8)	3.6 (29)
Moderate (2 factors)	31.4 (587)	28.9 (238)	29.7 (68)	34.5 (281)
CS	13.2 (246)	10.0 (82)	15.3 (35)	15.8 (129)
CK	6.9 (129)	8.3 (68)	5.3 (13)	5.9 (48)
KS	11.4 (212)	10.7 (88)	8.7 (20)	12.8 (104)
Expert (3 factors, CKS)	53.6 (1,000)	56.6 (466)	44.1 (101)	53.1 (433)

There was also a significant association between the extent of knowledge and survey type (*p* < 0.001), ecological zone (*p* < 0.001), gender (*p* = 0.001), age group (*p* < 0.001), and ethnicity (*p* < 0.001) of the participant. The extent of knowledge about the disease was lowest in individuals surveyed from clinical settings (34.0%), Semi-Urban settings, High Guinea Savannah zone, males, ≥60 years of age and Sudanese ethnic group, with only 44.7, 15.1, 47.6, 34.9, and 16.3%, respectively, fully knowledgeable about malaria.

### Adherence to national malaria control measures

**Figure 3:**
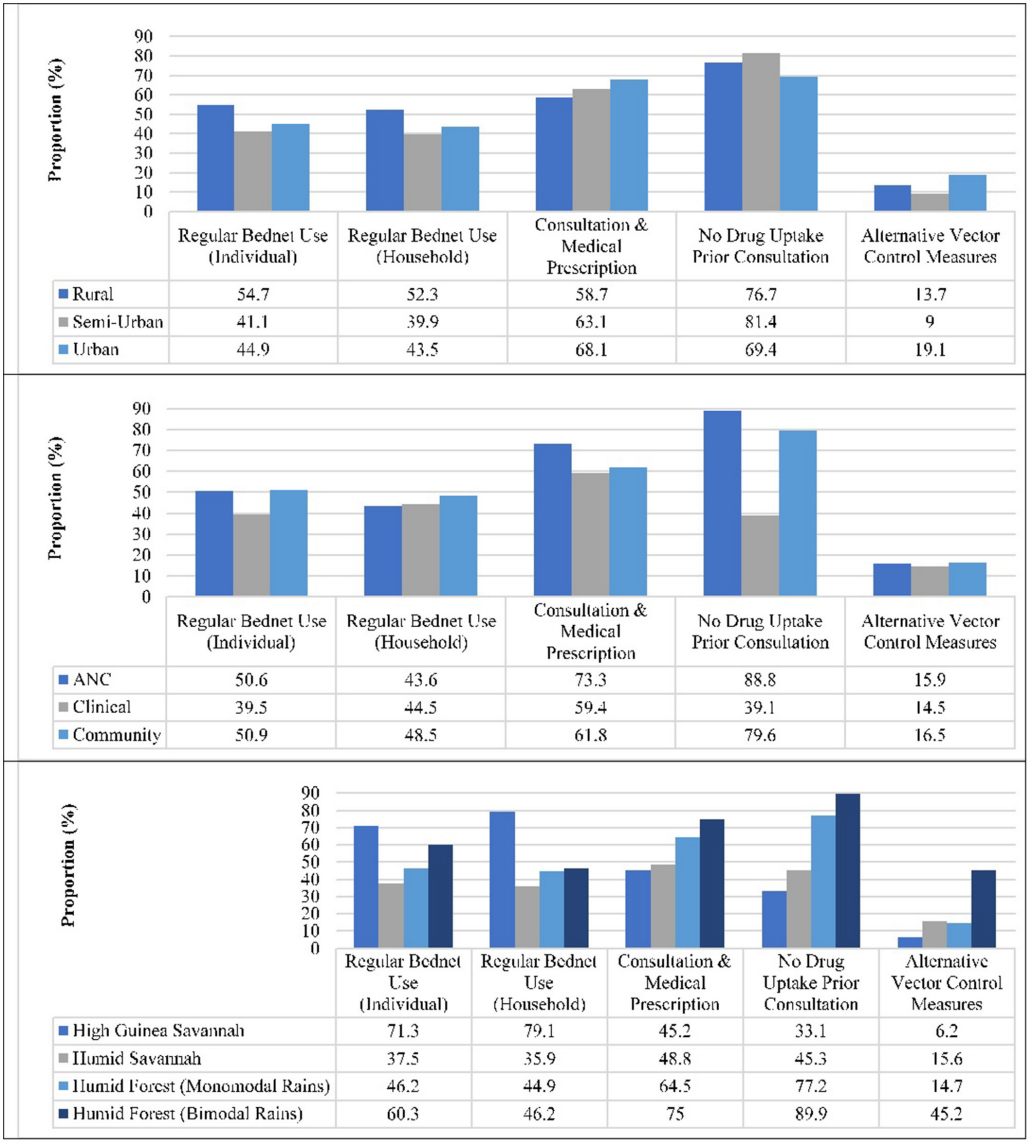
Positive attitudes and practices toward malaria control in Cameroon.

Almost half of the participants reportedly always used bednets at individual (48.5%), and household (46.9%) levels. Usage was significantly higher in participants from rural settings at both individual (55.6%, *p* < 0.001) and household (53.4%, *p* < 0.001) levels compared to their counterparts from Semi-Urban and Urban areas (Figure 3). Regular bednet usage at individual and household level was also highest (*p* < 0.001 each) in participants from the High Guinea Savannah (71.3 vs. 78.9%), and Sudanese ethnic group (69.5 vs. 77.4%), respectively. Over one in four participants reportedly took antimalarials prior to consultation, the level of auto-medication lower (*p* < 0.001) in Rural (23.3%) and Semi-Urban (18.6%) settings compared to their Urban counterparts (30.6%). The proportion of participants who took antimalarial prior consultation was also lowest in the Humid Forest zone with bimodal rainfall (9.3%, *p* < 0.001), pregnant women enrolled at ANC (11.2%), females (23.2%, *p* < 0.001), 15–20 year olds (20.4%, *p* = 0.035) and Bantu ethnic group (22.7%, *p* < 0.001).

Over sixty-three percent and 15.9% of participants reportedly adhered with the requirement for consultation prior to medical prescription and used alternative vector control measures, respectively. Nevertheless, the proportion of alternative vector control measures usage (*p* < 0.001) or consultation prior to medical prescription (*p* = 0.001) was higher in Urban settings (19.1 vs. 68.1%) compared to Rural (13.7 vs. 58.7%) and Semi-Urban (9.0 vs. 63.1%) settings (Figure 3). Majority of the participants from ANC (73.3%), Humid Forest zone with bimodal rainfall (72.2%, *p* < 0.001), females (66.1%, *p* = 0.001) and Bantu ethnic group (69.1% *p* < 0.001) adhered with the requirement for consultation prior to medical prescription. In addition, a higher proportion of individuals from Humid Forest zone with bimodal rainfall (46.5%, *p* < 0.001), males (18.4%, *p* = 0.040), 21–59 year olds (17%, 219/1,285, *p* = 0.038) and Bantu ethnic group (20.2%, *p* = 0.005) did take medication prior consultation. Alternative control strategies reportedly implemented against malaria include indoor residual spraying (4.9%), environmental sanitation (3.7%), burning of insecticide at bedtime (2.7%), seasonal malaria chemoprevention (1.3%), elimination of stagnant water (0.9%), shutting of doors and windows at dusk (0.7%), wearing of long-sleeved clothing (0.5%), netting of windows (0.2%) or use of a fan (0.2%).

A participant was classified as non-adherent, low, moderate or fully adherent if he/she reportedly respected none, at most two, 3–4 or all five malaria control measures, respectively. In all, only 2 (0.1%) of the 1,763 individuals interviewed were fully adherent and 105 (6.0%) non-adherent to malaria control measures while 1,025 (58.1%) and 631 (35.8%) were low and moderately adherent, respectively (Table 5). There was a significant association between the level of adherence and the locality of residence (*p* < 0.001) as well as with the survey type (*p* = 0.010), ecological zone (*p* < 0.001), and ethnicity (*p* < 0.001) of enrolled participants. The level of adherence to malaria control measures were highest in individuals enrolled from clinical settings, rural settings, the High Guinea Savannah zone and Sudanese ethnic group where 39.7, 38.5, 65.1, and 63.3%, respectively, were at least moderately adherent.

**Table 5:** Level of adherence to malaria control measures in Cameroon (*n* = 1,763).

**Level of adherence**	**All participants**	**Locality [% (** ***n*** **)]**			***P*-value**
	**[% (** ***n*** **)]**	**Rural**	**Semi-urban**	**Urban**	
None (0 factor)	6.0 (105)	4.2 31)	12.7 (28)	5.7 (46)	<0.001
Low (1–2 factors)	58.1 (1,025)	57.4 (425)	60.6 (134)	58.2 (466)	
Moderate (3–4 factors)	35.8 (631)	38.3 (284)	26.7 (59)	36.0 (288)	
Full (All 5 factors)	0.1 (2)	0.1 (1)	0.0 (0)	0.1 (1)	

### Malaria parasite infection and risk factors

A significant proportion of the population had asymptomatic parasitaemia amongst all surveyed participants (14.0%), pregnant women reporting to ANC (25.9%) and at community level (15.9%). Children 10–14 years (23.1 vs. 28.5%), non-adherents to malaria control measures (22.3 vs. 21.5%) and those with no knowledge of malaria (21.7 vs. 22.7%) were most infected overall and at community level, respectively (Table 6A). There was a significant association between the prevalence of asymptomatic malaria parasitaemia and the age group, level of adherence to malaria control measures and extent of knowledge about malaria of all study participants as well those enrolled through community surveys only (Table 6A). However, following multivariate analysis, age group, level of adherence to malaria control and extent of knowledge about malaria were identified as independent risk factors for asymptomatic malaria parasite infection in the study population overall as well as those enrolled through community surveys. Individuals aged 15–20 years were more likely to be infected when compared to those above 60 years of age in the study population overall (OR = 6.82, 95% CI 2.98–15.60, *p* < 0.001) as well in those from community surveys (OR = 8.26, 95% CI 3.25–21.02, *p* < 0.001) when compared to their counterparts of at least 60 years of age. Similarly, participants with moderate knowledge about malaria were protected from infection in the population overall (OR = 0.39, 95% CI 0.18–0.83, *p* = 0.014) as well as in those enrolled exclusively through community surveys (OR = 0.36, 95% CI 0.14–0.91, *p* = 0.031) when compared to their counterparts without knowledge of the disease. Individuals enrolled from Rural (OR = 2.73, 95% CI = 1.50–4.97, *p* = 0.001) and Semi-Urban (OR = 2.44, 95% CI = 1.16–5.14, *p* = 0.019) settings in community surveys were also more likely to be infected with the parasite when compared to Urban dwellers. Interestingly, individuals who were reportedly moderately adherent to malaria control measures were less likely to be infected with the malaria parasite in the population overall (OR = 0.44, 95% CI = 0.25–0.77, *p* = 0.004) as well as those from community surveys (OR = 0.36, 95% CI = 0.17–0.78, *p* = 0.009) relative to their non-adherent counterparts (Table 6A). Nevertheless, asymptomatic parasitaemia was independent of all risk factors assessed in pregnant women reporting at ANC.

**Table 6A:** Relationship between socio-demographic factors, adherence to malaria control, knowledge of malaria and asymptomatic malaria parasitaemia in Cameroon.

**Variable**	**Subgroup**	**All subjects**						**Community**					
		***N***	**% (** ***n*** **)**	**Unadjusted** ***P*****-value**	**OR**	**95 % CI**	**Adjusted** ***P-*****value**	***N***	**% (** ***n*** **)**	**Unadjusted** ***P-*****value**	**OR**	**95 % CI**	**Adjusted** ***P-*****value**
Age group (years)	15–20	2,048	19.8 (66)	<0.001	6.82	2.98–15.60	<0.001	1,212	27.0 (38)	<0.001	8.26	3.25–21.02	<0.001
	21–59		11.7 (178)		3.36	1.52–7.44	0.003		9.9 (92)		2.54	1.06–6.12	0.038
	≥60		5.3 (10)		Ref				6.3 (9)		Ref		
Locality	Rural	2,048	13.1 (110)	0.031	1.29	0.94–1.77	0.11	1,212	12.9 (101)	0.002	2.73	1.50–4.97	0.001
	Semi—urban		16.7 (39)		1.39	0.90–2.14	0.134		14.6 (23)		2.44	1.16–5.14	0.019
	Urban		10.7 (105)		Ref				5.5 (15)		Ref		
Gender	Female	2,048	14.1 (192)	0.001	1.78	1.28–2.49	0.00	1,212	11.2 (78)	0.724	0.96	0.65–1.44	0.856
	Male		9.0 (62)		Ref				11.8 (61)		Ref		
Level of adherence to malaria control	Full	1,838	0.0 (0)	0.006	0.56	0.33–0.93	0.026	1,102	0.0 (0)	0.015	0.50	0.25–1.01	0.054
	Moderate		10.9 (68)		0.44	0.25–0.77	0.004		8.8 (34)		0.36	0.17–0.78	0.009
	Low		13.7 (138)		0.00	0.00	0.999		12.4 (79)		0.00	0.00	0.999
	None		23.1 (24)		Ref				21.5 (14)		Ref		
Extent of knowledge of malaria	Expert	1,736	12.8 (126)	0.004	0.84	0.38–1.85	0.663	1,202	9.5 (64)	<0.001	0.96	0.37–2.47	0.926
	Moderate		10.9 (63)		0.39	0.18–0.83	0.014		9.9 (33)		0.36	0.14–0.91	0.031
	Limited		19.2 (41)		0.53	0.25–1.10	0.087		20.9 (31)		0.43	0.18–1.05	0.064
	None		21.3 (13)		Ref				22.2 (10)		Ref		

The overall prevalence of *Plasmodium* infection and clinical malaria was 45.0 and 29.6%, respectively. As expected, the proportion of malaria parasite infection was higher in participants from clinical surveys (65.8%, *p* < 0.001), those with fever at enrolment or history of fever (53.4%, *p* < 0.001) and anemic (52.1%, *p* < 0.001) compared to community surveys (32.6%), their afebrile (31.4%) and non-anemic (34.8%) counterparts.

At community level, malaria parasite infection was most prevalent in 15–20 years olds (42.7%), rural settings (25.7%) and individuals with no knowledge about malaria (33.3%) (Table 6B). Age group, locality of residence and extent of knowledge about malaria were independent risk factors for malaria parasite infection following multivariate analysis. Individuals aged 15–20 years (OR = 6.16, 95% CI = 3.10–12.26, *p* < 0.001) and 21–59 years olds (OR = 2.44, 95% CI = 1.32–4.51, *p* = 0.004) were more at risk of malaria parasite infection compared to their counterparts 60 years and above of age. Additionally, participants residing in rural settings (OR = 2.0, 95% CI = 1.35–2.95, *p* < 0.001) were more susceptible to infection compared to urban dwellers. Incidentally, the risk of infection was higher in participants with no (OR = 2.92, 95% CI = 1.35–6.35, *p* = 0.007) or limited (OR = 7.91, 95% CI = 1.20–3.02, *p* = 0.006) compared to their expertly knowledgeable counterparts.

**Table 6B:** Relationship between socio-demographic factors, adherence to malaria control, knowledge of malaria and malaria parasitaemia in individuals enrolled through community surveys in Cameroon.

**Variable**	**Malaria parasite infection**							**Malaria**					
	**Subgroup**	***N***	**% (** ***n*** **)**	**Unadjusted** ***P-*****value**	**OR**	**95 % CI**	**Adjusted** ***P-*****value**	***N***	**% (** ***n*** **)**	**Unadjusted** ***P-*****value**	**OR**	**95 % CI**	**Adjusted** ***P-*****value**
Age group (years)	15–20	1,225	42.7 (61)	<0.001	6.16	3.10–12.26	<0.001	1,212	16.3 (23)	0.017	2.50	0.99–6.30	0.052
	21–59		20.9 (196)		2.44	1.32–4.51	0.004		11.1 (103)		1.91	0.86–4.27	0.115
	≥ 60		11.7 (17)		Ref				5.6 (8)		Ref		
Locality	Rural	1,225	25.7 (203)	0.001	2.0	1.35–2.95	<0.001	1,212	13.0 (102)	0.002	1.36	0.85–2.20	0.202
	Semi—Urban		18.9 (30)		1.14	0.65–2.0	0.641		3.8 (6)		0.38	0.15–0.97	0.043
	Urban		14.9 (41)		Ref				9.6 (26)		Ref		
Gender	Female	1,225	22.5 (158)	0.916	1.04	0.77–1.41	0.796	1,212	11.3 (79)	0.719	1.10	0.74–1.63	0.653
	Male		22.2 (116)		Ref				10.7 (55)		Ref		
Level of adherence to malaria control	Full	1,115	50.0 (1)	0.265	0.83	0.45–1.53	0.553	1,102	50.0 (1)	0.191	1.98	0.69–8.70	0.208
	Moderate		19.8 (79)		0.70	0.36–1.32	0.268		11.4 (45)		1.95	0.66–5.81	0.229
	Low		23.6 (153)		3.98	0.22–70.86	0.347		11.4 (73)		17.03	0.91–352.3	0.057
	None		27.7 (16)		Ref				6.2 (4)		Ref		
Extent of knowledge of malaria	Expert	1,215	19.5 (133)	0.004	0.65	0.28–1.50	0.313	1,202	10.2 (69	0.643	0.55	0.17–1.74	0.308
	Moderate		22.8 (77)		0.43	0.20–0.94	0.033		12.9 (43)		0.72	0.25–2.04	0.531
	Limited		31.3 (47)		0.34	0.16–0.74	0.007		10.8 (16)		0.44	0.16–1.26	0.126
	None		33.3 (15)		Ref				11.1 (5)		Ref		

The prevalence of malaria was also highest in participants 15–20 years of age (16.3%) and in rural settings (13%) at community level. Locality of residence and extent of knowledge were identified as independent risk factors for malaria following multivariate analysis. Individuals enrolled from Semi-Urban settings were less at risk (OR = 0.38, 95% CI = 0.15–0.97, *p* = 0.043) while individuals with moderate knowledge were more susceptible (OR = 1.62, 95% CI = 1.05–2.49, *p* = 0.029) compared to their Urban and expertly knowledgeable counterparts, respectively (Table 6B).

In participants enrolled from ANC, only age group was a significant risk factor for both malaria parasite infection (OR = 1.84, 95% CI = 1.05–3.21, *p* = 0.033) and malaria (OR = 2.41, 95% CI = 1.31–4.45, *p* = 0.005) in pregnant women, following multivariate analysis. In this vulnerable group, individuals 15–20 years of age were more likely to be infected or harbor the disease when compared to their 21–59 year old counterparts.

## Discussion

The level of a population's knowledge about malaria, attitude, and adherence to control measures can affect the efficacy of interventions and effectiveness of strategies for the management and elimination of the disease. Poor adherence to malaria intervention measures, subject to individual attitudes, beliefs, resources and knowledge, may modulate the incidence and exacerbate the impact of the disease.

This study found a remarkably high level of knowledge about malaria and its management, with over three-quarters of the population surveyed either knowledgeable about the cause, symptoms or malaria control measures. This is consistent with previous reports of high awareness about malaria in the country. Accordingly, 82–91% of respondents in the cities of Douala and Yaounde (12, 35), and over 90% in four eco-epidemiological settings, Kaélé, Tibati, Bertoua, and Santchou (36) associated malaria to a mosquito bite, while 94.1% of respondents reportedly knew at least one symptom of the disease (35). However, the fact that just over half of the participants have expert knowledge about malaria in this study has implications for the control and management of the disease. This is because individual attitudes and practices against the disease are more likely to be shaped by overall knowledge and not dependent solely on a single aspect. The high level of awareness of malaria might be due to the perennial transmission and high disease endemicity/burden on victims rather than from organized formal educational and sensitization programs. Mainly school going children, pregnant women/nursing mothers who attend antenatal and post-natal clinics receive lectures or educational talks on cause, symptoms, effects and malaria control/prevention strategies. The rest of the population are only periodically educated on targeted strategies such as during bednet distribution campaigns, rendering them vulnerable to varied opinions.

Long lasting insecticidal nets, diagnosis and drugs have been the main malaria control interventions across Africa, and over two-thirds of the population surveyed here prioritized medical consultation/proper diagnosis/prescription or did not take medication prior consultation. This is in accordance with previous reports of the preference for hospital consultation by 63% of workers with malaria symptoms from enterprises in Douala (35). These practices are, however, heterogeneous across communities, with higher individual and household ITN usage in rural settings and in the High Guinea Savannah ecological zone. Other studies have reported 67.3% of participants in Buea (20) and 81.4% in Yaounde (12) reportedly practiced presumptive diagnosis and self-medication, respectively. However, the fact that less than half of the participants reportedly used bednets on a regular basis at individual and household is concerning.

Such low adherence to the use of LLINs, the most widely recommended malaria prevention strategy, has been attributed to climate change factors, resulting in high temperatures within homes. Some participants explain their reticence in using the tool to the associated heat and feeling of suffocation when under the bednet. Such behavioral patterns have been recurrent and reported in recent studies (37, 38). This has had multiplier effects resulting in very little self-care as the uncompromising feeling of heat among the majority low-income populations who are unable to afford fans or other air conditioning facilities leave them with no choice than sleeping in the heat but out of the ITNs. Conversely, a large proportion of the participants with no or torn bednets might justify the increase in the burden of the disease after several intervention strategies were employed.

Although knowledge of malaria should positively influence adherence to control measures, <1% of the participants in this study were fully adherent to malaria control measures, highlighting a significant discrepancy between knowledge of the disease and adherence (39). This is further supported by the lower individual/household bednet usage and higher proportion of self-diagnosis and uptake of antimalarials prior to medical consultation from urban dwellers who were more aware of the cause and symptoms of malaria. This suggests knowledge of the disease has a negative impact on adherence to control measures, with individuals probably relying on presumptive diagnosis to self-manage the disease. The reliance on self-management of malaria has been attributed to the inconvenience of pursing normal medical procedure, lack of means, easy access to drug stores or ambulant drug vendors, work pressure, fear of COVID-19 contamination or misdiagnosis and quarantine during the pandemic during which the surveys were undertaken and insufficient consideration for health care. Thus, knowledge of malaria must not necessarily lead to adherence to control measures unless supported by additional health system management measures (39, 40). For instance, malaria related consultation at health facilities and hospitalization for severe malaria increased in Buea health district following implementation of free malaria treatment services (40).

In a recent study in Douala by Nchetnkou et al. (35), for instance, only 77% of participants affirmed using bednets, even though 91% recognized the vector as the primary mode of malaria parasite transmission. Nevertheless, higher IPTp uptake has been reported in pregnant women reporting for antenatal care in Bamenda who were knowledgeable about the intervention (41). The fact IPTp-SP is provided for free to women at routine antenatal visits in Cameroon and educational/sensitization talks offered these women on the danger they and their child/children are exposed to if infected with malaria may explain the rate of adherence in these women and suggests that other facts, in addition to knowledge, are critical to the respect of the national malaria control guidelines and elimination of the disease.

With almost half of the surveyed participants infected with the parasite and 28.5% of children 10–14 years old harboring asymptomatic infection in communities in this study, the disease remains a significant health problem in the country. Age group, extent of knowledge of malaria and level of adherence to control measures were independent risk factors of asymptomatic malaria parasitaemia. Consistent with their increased parasite-specific immunity following repeated exposure over the years, participants 15–20 years were more at risk of asymptomatic malaria parasite carriage than their counterparts 60 years and above.

As would have been expected, participants with limited knowledge about malaria and those residing in rural/semi-urban settings were more likely to harbor the infection compared to their expertly knowledgeable and urban counterparts, respectively. In addition, even moderate adherence to malaria control strategies was associated with protection from the parasite. Rural communities in the area are characterized by factors that may interfere with national malaria control guidelines including houses with multiple openings/crevices in their wooden walls, sometimes without ceilings as well as sociological factors, such as, the tendency to always congregate outdoors or indoors late in the evenings, before bedtime, and sometimes beyond, presence of vegetation, poor drainage around settlements. These may increase exposure to mosquito bite and the likelihood of survival, growth and spread of *Plasmodium* spp. and stifling the fight against malaria.

The fact that almost half (46.5%) of the participants in the Humid Forest zone with bimodal rainfall reportedly took antimalarial medication prior to consultation is worrying. Self-medication may result to poor dosage, non-treatment of disease, wastage of resources, parasite adaptation and antimalarial resistance (42–44). This practice has been associated to mildness of symptoms, poverty (45), knowledge of symptom of the disease, proximity to drug vendors, and absence of health facility (20). This therefore underscores the need to continuously sensitize the population on the consequences of auto-medication and improved overall access and affordability of malaria treatment by the national malaria control program.

The study is limited by the fact that most participants were enrolled from the humid forest area (monomodal rainfall) while those in ANC and clinical groups were mostly from urban settings. This is likely to generate bias and suggest that the findings reported here may not reflect the heterogenous Cameroonian population. Nevertheless, we recommend the following: Education and sensitization programs on malaria in general should be organized on the media and other popular platforms targeting the segment of the population that are not opportune to attend formal education, while programs focusing on the risk of non-adherence to approved control measures should target mainly the educated mass. Enlargement and reinforcement of policies geared toward the implementation of free and compulsory diagnosis, treatment to everyone consulting at all health facilities and increase access to the facilities in addition to programmed periodic free malaria community screening and treatment.

## Conclusion

*Plasmodium* infection, asymptomatic parasitaemia and malaria remain a substantial public health concern in Cameroon. While a substantial proportion of the population was knowledgeable about malaria and exhibited some positive attitudes toward the control of the disease, full adherence to national malaria control strategies was negligible. The high prevalence of malaria parasitaemia suggests that more aggressive, robust and concerted measures at improving knowledge, attitudes and practices toward intervention strategies are necessary to curb the incidence of the infection and impact of the disease.

## Data availability statement

The raw data supporting the conclusions of this article will be made available by the authors, without undue reservation.

## Ethics statement

The studies involving human participants were reviewed and approved by Cameroon National Ethics Committee. Written informed consent to participate in this study was provided by the participants' legal guardian/next of kin.

## Author contributions

LT coordinated the study, performed field and laboratory experiments, and drafted the manuscript. MA performed field survey and drafted the manuscript. MM performed field surveys and provided substantial improvement in manuscript. HC, VNtu, PK, ET, MF, FF, JM, FTF, and VNta performed field surveys. TN and EY provided substantial improvement in manuscript. EA and AA-N contributed reagents and materials. TA conceived, designed and coordinated the study, performed the statistical analysis, and drafted the manuscript. All authors read and approved the final manuscript.

## Footnotes

^1^Cameroon Climate, Weather by Month, Average Temperature – Climate and Average Weather Year Round in Cameroon. Weather Spark. Available online at: https://www.weatherspark.com.

^2^Climate Details in Cameroon. Available online at: https://www.worlddata.info; Weather Atlas. Available online at: https://www.weatheratlas.com.
